# Efficacy and safety of non-ablative vaginal erbium:YAG laser treatment as a novel surgical treatment for overactive bladder syndrome: comparison with anticholinergics and β3-adrenoceptor agonists

**DOI:** 10.1007/s00345-019-02644-7

**Published:** 2019-01-28

**Authors:** Nobuo Okui

**Affiliations:** Uro-Gyn.Net Health Care Cooperation, Dr. Okuis’ Urogynecology and Urology Clinic, Ootaki 2-6, Yokosuka, Kanagawa 238-0008 Japan

**Keywords:** Non-ablative vaginal erbium:YAG laser, Overactive bladder syndrome, Anticholinergic agent, β3-Adrenoceptor agonist, Overactive bladder syndrome symptom score, Vaginal Health Index Scale

## Abstract

**Purpose:**

To examine the efficacy and safety of non-ablative vaginal erbium:YAG laser (VEL) for the treatment of overactive bladder syndrome (OAB) compared with those of two other common pharmacotherapies, namely, anticholinergics and β3-adrenoceptor agonists.

**Methods:**

Female subjects aged 60–69 years who presented with symptoms of OAB from 2015 to 2017 were assigned to three groups (*n* = 50) receiving treatment with an anticholinergic agent (4 mg fesoterodine), a β3-adrenoceptor agonist (25 mg mirabegron), or VEL (20 min/session of VEL performed thrice). The OAB symptom score (OABSS), Vaginal Health Index Scale (VHIS), and occurrence of adverse effects were examined prior to and at 1 year following treatment initiation.

**Results:**

The three groups showed significant improvement (*p *< 0.001) for all items of the OABSS questionnaire. Improved VHIS scores were observed only in the VEL group. Furthermore, after VEL treatment, a negative correlation was observed between questions 3 (urinary urgency) and 4 (urgency urinary incontinence) of the OABSS and VHIS. Regarding safety, no adverse events were observed in the VEL group. However, subjects in the other two groups complained of constipation, as indicated by the Constipation Assessment Scale scores, and mouth dryness. The therapeutic effects were inadequate for one and two subjects in the VEL and β3-adrenoceptor agonist groups, respectively.

**Conclusions:**

VEL safely and effectively improved OABSS through a different mechanism than that involved in pharmacotherapy. We propose the use of VEL as a novel surgical treatment option in the field of urology.

## Introduction

Overactive bladder syndrome (OAB), a syndrome characterized by the chief complaint of urinary urgency, is often accompanied by frequent urination, nocturia, and occasional urinary incontinence [1, 2]. This condition significantly reduces quality of life [2]. Pathologies and conditions underlying OAB are broadly divided into neurogenic (caused by neurological diseases including brain and spinal diseases) and non-neurogenic (absence of clear neurological disease). Non-neurogenic causes of OAB vary substantially, including aging and pelvic organ prolapse [3]. In non-neurogenic OAB studies, considerable bladder ischemia has recently drawn attention [4].

Pharmacotherapy is the main modality used for OAB treatment. There are two types of pharmacotherapies: anticholinergics and β3-adrenoceptor agonists [5]. However, these cannot be administered to all patients with OAB owing to the occurrence of adverse effects [6]. Anticholinergic drugs block the muscarinic receptor in the whole body, possibly leading to the development of adverse effects on the central nervous system [7]. β3-Adrenoceptor agonists may cause increased risk of contraindications in patients with severe uncontrolled hypertension because it can exacerbate this condition [8].

Non-ablative vaginal erbium:YAG laser (VEL) is considered safe and effective [9]. VEL improves blood flow in the region surrounding the vagina and promotes tissue reconstruction [10] and has a favorable safety profile [9, 11].

Therefore, it is worth examining the effects of VEL, anticholinergics, and β3-adrenoceptor agonists on OAB treatment. Previous studies have reported the effects of urinary urgency and urgency urinary incontinence on mixed incontinence [11], compared VEL and treatment with estriol, and investigated the effect of VEL alone in OAB treatment [12]. However, there are no comparative studies on treatments used for OAB [13]. We believe that this study is the first to prospectively compare VEL and pharmaceutical agents for OAB treatment.

## Subjects and methods

### Selection of subjects

The study population included females aged 60–69 years who presented with symptoms of OAB at Dr. Okuis’ Urogynecology and Urology (Kanagawa, Japan) between 2015 and 2017. The diagnosis was determined according to the Japanese guidelines [14] requiring ≥ 2 points for question (Q) 3 (indicating urinary urgency) of the OAB symptom score (OABSS) questionnaire, which is commonly used in the field of urology [14, 15]. Moreover, the diagnosis was based on a medical interview, water intake habit, physical/neurological findings, residual urine, urine analysis, medical history, history of present illness, and complications. The study inclusion criteria included females not undergoing OAB treatment and those without a history of female hormone replacement therapy or botulinum toxin injection therapy. The presence/absence of brain, spinal, and peripheral nerve diseases was verified, and patients with neurogenic OAB were excluded. Patients with gynecological diseases in the adjacent organs (e.g., uterine cancer) were also excluded. However, the presence/absence of age-related vaginal changes was not considered. Further, pelvic organ prolapses (POP) were measured by POP system [16]. Females with POP stage 0 and those with a history of surgery for the same were excluded [3]. Patients with stress incontinence were excluded. In the event of occurrence of adverse effects during treatment affecting daily life, the treatment was discontinued. During the observation period, treatment with other therapies for OAB was not permitted. The subjects in the VEL, anticholinergic, and β3-adrenoceptor agonist (β3) groups were enrolled in a medical chart number-based sequential order.

### Treatment with VEL

After spraying the vagina of the subjects with 9% xylocaine, the tip of FotonaSmooth™XS (Fotona d.o.o., Ljubljana, Slovenia) was inserted into the vagina. Using the 2940 nm VEL with the proprietary “long pulse” setting, laser energy was applied to the complete anterior vaginal wall for 10 min and to the complete vagina for 10 min [13]. Laser energy was applied once per month for 3 months. Follow-up observation was performed for 1 year, with day 0 being the day of first administration of treatment.

The United States Food and Drug Administration (FDA) issued a warning in 2018 regarding the application of laser energy to the vagina [17]. We selected an authorized device that did not fall under the FDA’s warning; we monitored the presence or absence of adverse effects through regular interaction once annually with the study subjects even after study completion.

### Information pertaining to the administration of therapeutic agents

The drug dosage was administered according to the Japanese guidelines [15]. In the anticholinergic and β3 groups, 4 mg fesoterodine and 25 mg mirabegron were administered once daily after breakfast, respectively. Fesoterodine was selected as a drug against OAB that was to be orally administered; it was used widely and was new in Japan [18]. Adopted criteria were the subjects with 95% and more treatment adherence. In the event of occurrence of a severe adverse event, treatment was discontinued and 1-year follow-up observation was performed, with day 0 being the day of first administration of treatment. Every patient and their families met with a doctor and nurse every month and received guidance on diet, water intake, sleep, exercise, etc.

### Evaluation of efficacy and safety

The therapeutic outcomes were evaluated using the OABSS questionnaire [11]. The OABSS questionnaire was completed during the initial examination and at 1 year following treatment initiation. Residual urine was measured through ultrasound. The Vaginal Health Index Scale (VHIS) was used [19] and safety was examined. For VHIS, a system widely used in the field of gynecology, the appearance of vaginal epithelium (elasticity, pH, vaginal discharge, and epithelial integrity and moisture) was evaluated via internal examination performed by a physician. Each parameter was graded on a scale of 1–5 (1 indicating no elasticity and 5 indicating excellent elasticity), with a total score of ≤ 15 indicating an atrophic condition. Constipation was evaluated using the Constipation Assessment Scale (CAS) [20], including eight items scored on a scale of 0–2 points, with a higher score indicating severe constipation. The degree of mouth dryness was evaluated according to a five-point scale (1 indicating extremely good and 5 indicating extremely bad). All other adverse events were recorded by free description. All treatments and examination were administered by a single physician accompanied by professional nurses. The pill counts were not used and the adherence to medication was assessed via interviews with the patient and the family.

### Education program

Without education, some patients in the medication group would have dropped out. Conversely, it has been easily predicted that there are no incidences of dropout among patients in the VEL group [16]. We established clinically effective education. Professional nurses and I have a meeting with the specialist to provide all patients and their families with an opportunity to participate in education programs every month. In the LIDRE Medical Center, there are all types of specialist physicians. At the first meeting, we explained the purpose and research methodology to the patients and their families, including their daughter, sister, or husband, using our health guide book. In our books, information on appropriate water intake, sleep, ideal meal, and exercise has been provided. The book is available for sale in Japan. Clinical trials education for Japanese may show high effects. Yoshida conducted a study on the B3-adrenorecepor agonist Vibegron and only 11 patients discontinued treatment out of the 372 patients in Vibegron 50 mg group [21]. To avoid a strong placebo effect in the OAB study, the VEL and pharmacotherapy groups received identical teaching/counseling regarding lifestyle modification.

### Statistical analysis

Statistical significance was evaluated using Student’s *t* test. The Pearson correlation coefficient was used to determine the correlation coefficient. Statistical analysis was performed using Microsoft Excel 2016 (Microsoft, Redmond, Washington, USA). *P* value < 0.001 was considered statistically significant.

## Results

### Study subjects

We orally acquired the patients’ clinical situation from the patients and their families. The education provided by us showed a high level of adherence. There were no incidences of dropout among the patients. Adherence to oral OAB medications was 97.8 ± 1.80% at 1 year. Table 1 presents the basic pretreatment data for the three groups. Before the treatment, there were no significant differences observed in terms of age, duration of OAB, number of deliveries, and VHIS scores among the three groups. One year later, change of treatment was desired by one patient in the VEL group (insufficient treatment effect), two in the anticholinergic group (mouth dryness), and two in the β3 group (insufficient treatment effect). The 1-year persistence in the anticholinergic and β3 groups were 96.6 ± 1.47% and 98.9 ± 1.30%, respectively.

**Table 1:** Basic characteristics of the three groups

	VEL group	Anticholinergics group	β3 group	Normal level
No. of enrolments	50	50	50	
No. of individuals observed for 1 year	50	50	50	
Age at beginning of the treatment (years)	63.8 ± 2.56	63.9 ± 2.76	65.32 ± 2.28	
OAB disease duration (years)	2.68 ± 0.81	2.41 ± 0.67	2.75 ± 1.74	
No. of deliveries	1.98 ± 1.02	1.7 ± 0.7	1.56 ± 0.64	
Body mass index (kg/m^2^)	24.9 ± 1.38	25.3 ± 1.59	25.2 ± 1.38	
Maximum blood pressure (mmHg)	115.5 ± 16.4	114.5 ± 18.4	116.0 ± 14.3	
Lowest blood pressure (mmHg)	80.3 ± 12.8	81.1 ± 13.0	80.0 ± 14.4	
Blood glucose (mg/dL)	98.1 ± 16.0	97.0 ± 18.0	99.0 ± 17.2	Before load 70–109
Hemoglobin A1c (%)	5.5 ± 0.29	5.5 ± 0.31	5.5 ± 0.32	4.6–6.2
Hemoglobin (g/dL)	13.3 ± 0.62	13.4 ± 0.69	13.3 ± 0.57	11.2–15.2
Hematocrit (%)	33.9 ± 12.3	34.6 ± 12.0	35.1 ± 11.5	34.3–45.2
Total protein (g/dL)	9.8 ± 6.8	9.8 ± 5.6	9.4 ± 6.2	6.5–8.2
Glutamic oxaloacetic transaminase (U/L)	21.1 ± 5.09	20.7 ± 5.13	21.4 ± 5.4	10–40
Blood urea nitrogen (mg/dL)	16.2 ± 3.0	16.6 ± 3.5	16.2 ± 3.2	8.0–20.0
Creatinine (mg/dL)	0.5 ± 0.1	0.5 ± 0.1	0.5 ± 0.1	0.46–0.82
No. of individuals with ≥ 100 ml residual urine	0	0	0	
No. of individuals who desired other treatment after completion of the observation period	1	2	2	
VHIS scores	10.52 ± 1.27	10.34 ± 0.84	10.18 ± 0.94	

### OABSS

**Fig. 1:**
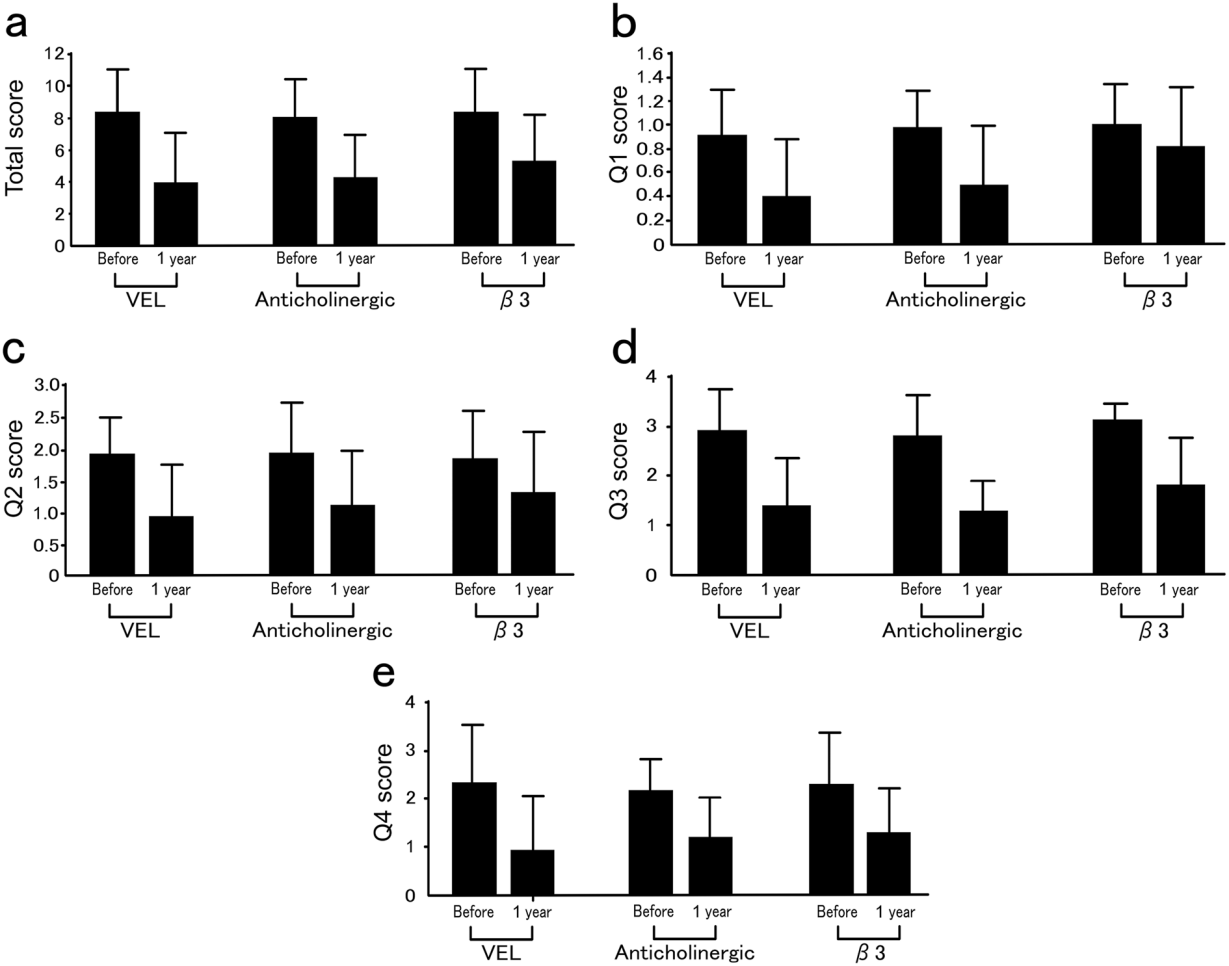
The OABSS results of the three groups. **a** The total OABSS scores are shown. The patients were classified into three groups. **b** Scores for Q1 of the OABSS, **c** scores for Q2, **d** scores for Q3, **e** scores for Q4

In Fig. 1a–e, the *X*-axis indicates the results prior to treatment and 1 year following treatment initiation in the three groups, whereas the *Y*-axis indicates the individual scores in each group.

For each item below, data prior to and at 1 year following treatment initiation are shown. Overall, the OABSS after the treatment significantly improved compared with the pretreatment scores in all groups (*p *< 0.001).

In the VEL, anticholinergic, and β3 groups, the scores improved from 8.16 ± 2.86 to 3.76 ± 3.30, 7.96 ± 2.49 to 4.16 ± 2.59, and 8.30 ± 2.88 to 5.25 ± 3.08, respectively (Fig. 1a).

For Q1 indicating frequent urination, the OABSS significantly improved in all groups (*p *< 0.001). In the VEL, anticholinergic, and β3 groups, the scores improved from 0.92 ± 0.39 to 0.40 ± 0.49, 0.98 ± 0.32 to 0.50 ± 0.51, and 1.00 ± 0.34 to 0.80 ± 0.53, respectively (Fig. 1b).

For Q2 indicating nocturia, the OABSS significantly improved in all groups (*p *<0.001). In the VEL, anticholinergic, and β3 groups, the scores improved from 1.94 ± 0.59 to 0.94 ± 0.84, 1.94 ± 0.82 to 1.12 ± 0.87, and 1.86 ± 0.76 to 1.32 ± 0.96, respectively (Fig. 1c).

For Q3 indicating urinary urgency, the OABSS significantly improved in all groups (*p *< 0.001). In the VEL, anticholinergic, and β3 groups, the scores improved from 2.94 ± 0.89 to 1.44 ± 0.99, 2.86 ± 0.85 to 1.32 ± 0.65, and 3.12 ± 0.36 to 1.80 ± 0.99, respectively (Fig. 1d).

For Q4 indicating urgency urinary incontinence, the OABSS significantly improved in all groups (*p *< 0.001). In the VEL, anticholinergic, and β3 groups, the scores improved from 2.36 ± 1.26 to 0.98 ± 1.15, 2.22 ± 0.67 to 1.22 ± 0.86, and 2.32 ± 1.11 to 1.32 ± 0.96, respectively (Fig. 1e).

### VHIS

Data prior to and at 1 year following treatment initiation are shown below. There was no significant difference in terms of preoperative VHIS scores among the groups. Following the treatment, a significant improvement was observed only in the VEL group (*p *< 0.001). In the VEL, anticholinergic, and β3 groups, the VHIS scores improved from 10.52 ± 1.27 to 19.96 ± 1.01, 10.34 ± 0.84 to 10.26 ± 0.92, and 10.18 ± 0.94 to 10.2 ± 0.80, respectively. In the VEL group, the OABSS for Q3 and Q4 after the treatment showed a negative correlation with the VHIS at − 0.50 and − 0.46, respectively. There was no improvement in one patient in the VEL group (insufficient therapeutic effect). In the other groups, there was no correlation detected prior to and at 1 year after treatment initiation between the VHIS scores and each question of the OABSS questionnaire.

### Safety

Data prior to and at 1 year following treatment initiation are shown below. Increased residual urine was not reported by any of the subjects. Regarding the CAS indicating constipation (< 16 points), in the VEL, anticholinergic, and β3 groups, the scores improved from 3.0 ± 0.72 to 3.05 ± 0.79, 2.95 ± 0.69 to 4.05 ± 2.11, and 2.95 ± 0.68 to 3.2 ± 1.28, respectively. The difference was significant only in the anticholinergic group (*p *< 0.001). Overall, CAS scores of < 4 points before the treatment but of ≥ 8 points at 1 year following treatment initiation were observed in five and two patients in the anticholinergic and β3 groups, respectively. Before the treatment, there was no occurrence of mouth dryness (maximum: 5 points) in any group. However, after the treatment, 11 and 2 patients in the anticholinergic and β3 groups, respectively, had scores of ≥ 3 points. In the VEL group, there was no occurrence of adverse events. There were no abnormalities observed in blood pressure, electrocardiography, blood count, biochemistry, and urine analysis in any of the groups.

## Discussion

Regarding the therapeutic effect, improvement was noted in all the groups as determined by the OABSS questionnaire. Adverse effects were observed in the pharmacotherapy groups. Conversely, no adverse effects were reported in the VEL group. Based on these findings, we conclude that the occurrence of adverse effects should be considered during examination.

Several patients receive long-term anticholinergic-based treatment. However, prolonged exposure to these agents is associated with the development of adverse effects. Anticholinergics are also used as antidepressants and medication against Parkinson’s disease. Studies have shown that prolonged exposure (i.e., 20 years) strongly correlated with an increased risk of developing dementia [22]. This has led to the development of anticholinergics with a favorable safety profile and β3-adrenoceptor agonists with a different mechanism of action. However, the effect of prolonged exposure to these drugs is currently unknown. Thus, caution should be exercised when using them aiming at administering the minimum effective dose.

Fesoterodine is commercially available in Japan in doses of 4 and 8 mg. It has high bladder selectivity [23] and limited effect on the central nervous system [24]. In a trial directly comparing low (4 mg) and high (8 mg) doses of fesoterodine, statistically significant dose-dependent effects were observed [25]. However, the occurrence of adverse effects may be associated with increase in dose [26]. In this study, mouth dryness led to the desire to change the treatment in 11/50 patients (22%).

Mirabegron is another commercially available medication in Japan used in 25 and 50 mg doses. The incidence of adverse events following its use is low. Despite the satisfactory treatment effects observed even with a low dose (25 mg), the administration of mirabegron has been linked to increase in blood pressure (2.8% of patients) and residual urine (2.8%). Moreover, treatment discontinuation was desired by 4.8% and 16% of patients due to adverse and insufficient treatment effects, respectively [27]. A previous study reported that the occurrence of adverse effects was not dependent on dose [2]. In this study, the occurrence of mouth dryness was similar (4% and 0.9–1.8% at doses of 25 and 50 mg, respectively). Although combination therapy of mirabegron with anticholinergics has advantages [26], it is not indicated in all the patients. Based on these findings, VEL seems to be a novel treatment option for OAB.

VEL has demonstrated comparable efficacy to those reported for 4 mg fesoterodine and 25 mg mirabegron. It improves blood flow in the bladder, urethra, and vaginal wall [10, 11]. However, it is considered ineffective for the treatment of neurogenic OAB. Moreover, the subject population was limited to those with non-neurogenic OAB. The effects were comparable to those reported with the use of pharmacotherapy, indicating that VEL may not necessarily be effective in all the patients with OAB. However, depending on its use, it carries the potential to fundamentally change the treatment of OAB.

VEL was the only treatment to effectively improve vaginal health status as shown by the VHIS scores, which is consistent with the results of a previous study that reported that VEL promotes vaginal cell synthesis and improves the VHIS scores [19]. In the report on the genitourinary syndrome of menopause, which is a new definition for the variety of menopausal symptoms (GMS) associated with physical changes of the vulva and vagina, as described by Gambacciani [19], 205 GMS women (mean 61.2 ± 7.2 years) showed improvement from 10.6 ± 2.7 to 16.5 ± 2.0 in the VHIS score after 1 year with VEL. The baseline and post-improvement scores are almost the same as reported in this study. They also showed improvement in voiding symptoms after VEL. It is possible that the baseline VHIS scores of patients with OAB are similar. In addition, the vaginal condition after the treatment correlated with Q3 and Q4 of the OABSS questionnaire. Therefore, the results suggest that the effects of VEL treatment and pharmacotherapy are exerted through different approaches. Consequently, considering the mechanism of VEL is necessary when examining its role in the treatment of OAB.

In the VEL group, the relationship between the vaginal condition and OAB, from a different perspective, can be considered a pathway that closely connects the vagina and bladder via the OAB mechanism.

Impairment of the bladder blood flow has been suggested as the mechanism involved in OAB onset [28]. In an experiment involving rabbits, endothelial dysfunction of the iliac artery caused impairment of the bladder wall blood flow, which led to detrusor overactivity. Further impairment of blood flow caused contractile dysfunction due to bladder wall fibrosis [29]. Follow-up studies of blood flow impairment and OAB have been performed using various animal models [30–33]. The use of free radical scavengers in ischemia has recently drawn attention for the treatment of OAB [34].

Therefore, we believe that VEL can improve OAB through a different mechanism to that involved in pharmacotherapy. Thus, various applications of VEL may be expected in the future, such as treatment of non-neurogenic OAB, combination therapy to reduce drug dosage, and combination with pharmacotherapy for patients with both neurogenic and non-neurogenic OAB.

This study has some limitations. First, this study was conducted at a single hospital and the patients were observed by a single doctor. Second, the pill counts were not used. These led to the study being assessed as that with poor objectivity. Third, there was no placebo arm in the study. To compensate for this, every patient received exactly equal education. Fourth, the education program may have affected the results. In general practice, the 1-year persistence to oral OAB medications was 57–72% for Korean patients [35]. In a previous study on antimuscarinics and mirabegron, the 1-year persistence was broadly 12–25% and 32–38%, respectively [36]. The 1-year persistence of this study was 97.8% (total), 96.6% (anticholinergic) and 98.9% (mirabegron). For these limitations, my practice was significantly different from general practice.

## Conclusion

The use of VEL, anticholinergics, and β3-adrenoceptor agonists improved OAB. VEL exhibited a favorable safety profile (no adverse effects) and possibly involves a different mechanism of action to that observed following the administration of drugs. Therefore, VEL may be a novel treatment option for OAB.
